# Reducing global air pollution: the scope for further policy interventions

**DOI:** 10.1098/rsta.2019.0331

**Published:** 2020-09-28

**Authors:** Markus Amann, Gregor Kiesewetter, Wolfgang Schöpp, Zbigniew Klimont, Wilfried Winiwarter, Janusz Cofala, Peter Rafaj, Lena Höglund-Isaksson, Adriana Gomez-Sabriana, Chris Heyes, Pallav Purohit, Jens Borken-Kleefeld, Fabian Wagner, Robert Sander, Hilde Fagerli, Agnes Nyiri, Laura Cozzi, Claudia Pavarini

**Affiliations:** 1 International Institute for Applied Systems Analysis, IIASA, A-2361 Laxenburg, Austria; 2 Institute of Environmental Engineering, University of Zielona Góra, Zielona Góra, Poland; 3 Norwegian Meteorological Institute (met.no), Oslo, Norway; 4 International Energy Agency (IEA), Paris, France

**Keywords:** global air pollution, emission scenarios, policy scenarios, health impacts

## Abstract

Over the last decades, energy and pollution control policies combined with structural changes in the economy decoupled emission trends from economic growth, increasingly also in the developing world. It is found that effective implementation of the presently decided national pollution control regulations should allow further economic growth without major deterioration of ambient air quality, but will not be enough to reduce pollution levels in many world regions. A combination of ambitious policies focusing on pollution controls, energy and climate, agricultural production systems and addressing human consumption habits could drastically improve air quality throughout the world. By 2040, mean population exposure to PM2.5 from anthropogenic sources could be reduced by about 75% relative to 2015 and brought well below the WHO guideline in large areas of the world. While the implementation of the proposed technical measures is likely to be technically feasible in the future, the transformative changes of current practices will require strong political will, supported by a full appreciation of the multiple benefits. Improved air quality would avoid a large share of the current 3–9 million cases of premature deaths annually. At the same time, the measures that deliver clean air would also significantly reduce emissions of greenhouse gases and contribute to multiple UN sustainable development goals.

This article is part of a discussion meeting issue ‘Air quality, past present and future’.

## 1. Background

### (a) Context

The current exposure to air pollution in ambient air has been identified as the worldwide largest environmental risk factor for human health [1]. Anthropogenic activities emerge as the main drivers for emissions of air pollutants and add to pre-existing sources of natural emissions (soil dust, sea salt, vegetation, etc.) [2]. Human development affects emissions along multiple pathways: increasing pressure from population growth, industrialization and modern lifestyles is counteracted by technological progress, structural changes in the economy and targeted pollution control efforts. The interplay of these factors is changing over time; while pollution levels in industrialized countries have decreased, the developing world witnesses unprecedented levels of pollution today. The World Health Organization (WHO) estimates that currently about 90% of the people living in cities are exposed to PM2.5 levels above the WHO guideline value of 10 µg m^−^³, and globally between 3 and 9 million cases of premature deaths annually have been attributed to exposure to ambient air pollution [1,3,4].

Given the dynamics of these factors and their complex interplay, what could be expected for future air quality around the world, and which determinants will be dominating? To answer this question, this paper identifies key factors that contributed to historic air pollution trends in different world regions, outlines conceivable ranges of their future development and examines their interplay on global air quality in the next decades. In particular, the paper provides a fresh perspective on how ambitious policy interventions could achieve clean air worldwide.

### (b) Connecting air pollution and development

#### (i) Past trends

The observed increase and subsequent decline in SO_2_ emissions in many industrialized countries during the second half of the twentieth century inspired the ‘environmental Kuznets curve’ hypothesis, suggesting that environmental degradation tends to get worse as modern economic growth occurs until average income reaches a certain point [5,6]. However, the explanatory power and general validity of this hypothesis has been strongly contested [7–12], *inter alia,* because similar turning points have not yet been observed for other substances, including agricultural ammonia (NH_3_) and greenhouse gas emissions. Also, the role of (environmental) policy interventions is not explicitly recognized but implicitly subsumed as an autonomous concomitant of economic growth.

Rafaj *et al.* [13] identified three key factors that contributed to the observed decoupling between economic activity and air pollution in Europe between 1960 and 2010: (a) economic structural change, i.e. conversion to less energy-intensive economic activities, (b) energy policy, i.e. phase-out of oil and coal, and (c) dedicated air pollution control policies requiring efficient end-of-pipe cleaning devices (e.g. flue gas desulfurization, catalytic converters). As a result, during a period in which GDP quadrupled, SO_2_ emissions in Europe declined by 90% and NO_x_ emissions returned to their 1960 levels after an increase by a factor of three. Similar findings are reported by [14] for Europe and by [15] for North America. Most recently, decoupling of economic development trends and SO_2_ and NO_x_ emissions and the critical role of policy interventions have been observed in China [16–18], but not yet in India [19].

#### (ii) Future projections of air pollutant emissions

A range of studies in the scientific literature explored the implications of these findings on future emissions and air quality. For a long time, future global air pollutant trends were mainly modelled in the context of long-term greenhouse gas emission scenarios [20]. The early global studies on air pollutant emissions, notably the scenarios developed for the ‘Special Report on Emissions Scenarios' [21] and the ‘Representative Concentration Pathways’ [22,23] that have been prepared for the Intergovernmental Panel on Climate Change (IPCC) proposed declining trends of (energy-related) air pollutants, due to autonomous technological progress and assumed pollution control policies along the environmental Kuznets hypothesis. Later, the improved understanding of the importance of targeted air quality policy interventions motivated a more differentiated approach to projections of air pollutant emissions, resulting in a wider range of air pollutant trajectories than in previous global scenarios [24–26]. At the same time, the climate community addressed the interactions between decarbonization strategies and air pollutant emissions, both with the interest to reveal health benefits from low carbon policies [24,27–31]) and to explore the combined impacts of long-lived greenhouse gases and short-lived air pollutants (e.g. SO_2_ and black carbon) on radiative forcing and temperature increase [32–34]. In general, the literature reveals strong impacts of ambitious decarbonization strategies on energy-related air pollutants SO_2_, NO_x_ and PM, due to the phase-out of fossil fuels and the containment of all flue gases connected with carbon capture and storage. However, enhanced use of biomass as a greenhouse gas policy measure may lead to higher PM emissions [35–38].

Compared to the climate-focused analyses that deal mainly with energy-related emissions and the role of climate policy interventions, only a few studies addressed the longer-term prospects for air pollution from a health- and ecosystems perspective [4,39,40]. These studies take full account of other sources that also contribute substantially to population exposure to harmful air pollution, such as agricultural activities, waste management and materials handling. Also, they developed a more holistic approach towards the understanding of future trends in nitrogen emissions and their health and environmental impacts.

Not so many studies address air pollution at the global scale [26,41,42]. This is not surprising as, due to its physical features, air pollution is often considered as a local/regional and short-term problem, even if it is of universal nature, i.e. occurring as a concomitant of development in most industrialized areas throughout the world. However, a global perspective is of interest due to the intercontinental transport of pollution [43,44], the serious global health burden of air pollution [1] and the intimate interactions between clean air policies and efforts to achieve the UN global sustainable development goals [45–47].

For the second half of the twenty-first century, most long-term studies are in general agreement about declining energy-related air pollutant emissions; greenhouse gas mitigation strategies would accelerate the decline. However, projections differ for the next few decades, and particularly for the timing of global emission peaks.

## 2. Method

To explore the conceivable range of future air quality and, in particular, of population exposure to PM2.5 which has been associated with the most harmful health impacts [48], this paper develops a series of alternative emission scenarios up to 2040. Building on a widely accepted economic growth path with its structural economic changes, these scenarios combine different assumptions on the key policy areas that have been identified as critical for air pollution trends in the past, i.e. (a) energy/climate policy, (b) agricultural policies and (c) dedicated pollution control policies.

The analysis employs the greenhouse gas-air pollution interactions and synergies (GAINS) model [49]—see electronic supplementary material. For exogenous projections of future emission-generating human activities (i.e. energy, transport, industrial production, agricultural activities and waste volumes), GAINS computes emissions of 10 air pollutants and six greenhouse gases (see electronic supplementary material) considering the current emission characteristics in 180 regions. For the future, GAINS takes account of the penetration of already decided control measures and estimates the potential for additional emission reductions that is offered by several hundreds of control measures. The resulting emissions of all precursor emissions of PM2.5 in ambient air, i.e. primary PM2.5, SO_2_, NO_x_, NH_3_ and volatile organic compounds (VOCs), are then fed into an atmospheric dispersion model to compute annual mean concentrations of PM2.5 across the globe. GAINS employs reduced-form source-receptor relationships that have been derived from the EMEP atmospheric chemistry-transport model [50] with a spatial resolution of 0.125° × 0.0625° (approx. 7 km × 7 km) in Europe [51] and 0.1° × 0.1° (approx. 5–10 km × 5–10 km, depending on latitude) outside Europe, distinguishing about 6000 individual cities with more than 100 000 inhabitants. Resulting concentration fields are then compared with air quality standards, and corresponding population exposure is computed for the population distribution assumed in the socio-economic projection. More detail is given in the electronic supplementary material.

## 3. Results

### (a) Historic emission trends

**Figure 1.**
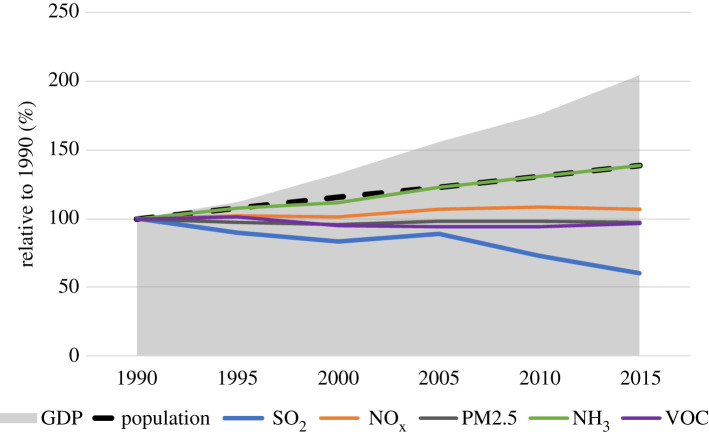
Trends of global GDP, population and precursor emissions of PM2.5 from anthropogenic sources, 1990–2015 (relative to 1990) (source GAINS). (Online version in colour.)

Over the last 25 years, SO_2_ emissions from anthropogenic activities declined by 40% at the global level. Primary emissions of PM2.5 and of VOC stabilized, NO_x_ grew by about 10% and NH_3_ by one-third (figure 1). Obviously, these global trends emerge as an aggregate of sometimes diverging regional trends (see §3c(iii)).

**Figure 2.**
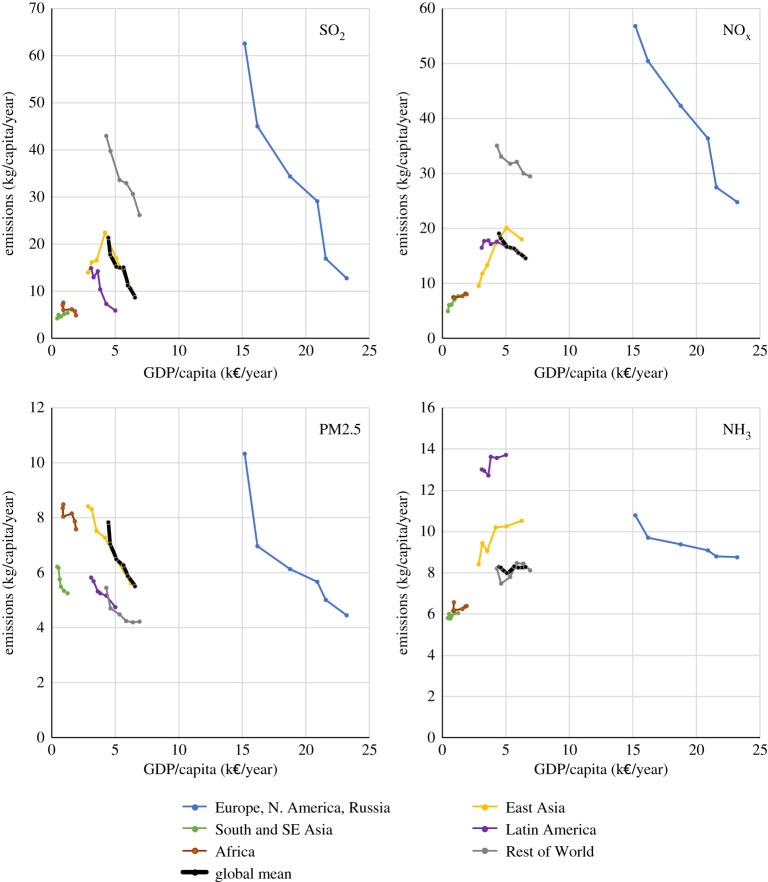
*Per capita* emissions of PM precursors versus *per capita* income from 1990 to 2015 (in 5-year steps), for the six world regions (source GAINS). (Online version in colour.)

During the same period, GDP doubled, and world population grew by more than a third, i.e. faster than most of the air pollutant emissions. Thus, except for NH_3_, emissions on a *per capita* basis declined globally in these 25 years, in line with the earlier observations that led to the formulation of the environmental Kuznets hypothesis [5,6] (figure 2). It is noteworthy that after 1990 clear peaks in *per capita* emissions could only be observed for SO_2_ and NO_x_ in China, while all other world regions exhibit constant or declining trends following the increase in *per capita* income. Also, *per capita* emissions of the six world regions that showed striking differences in the past reduced over time despite large disparities in income levels.

**Figure 3.**
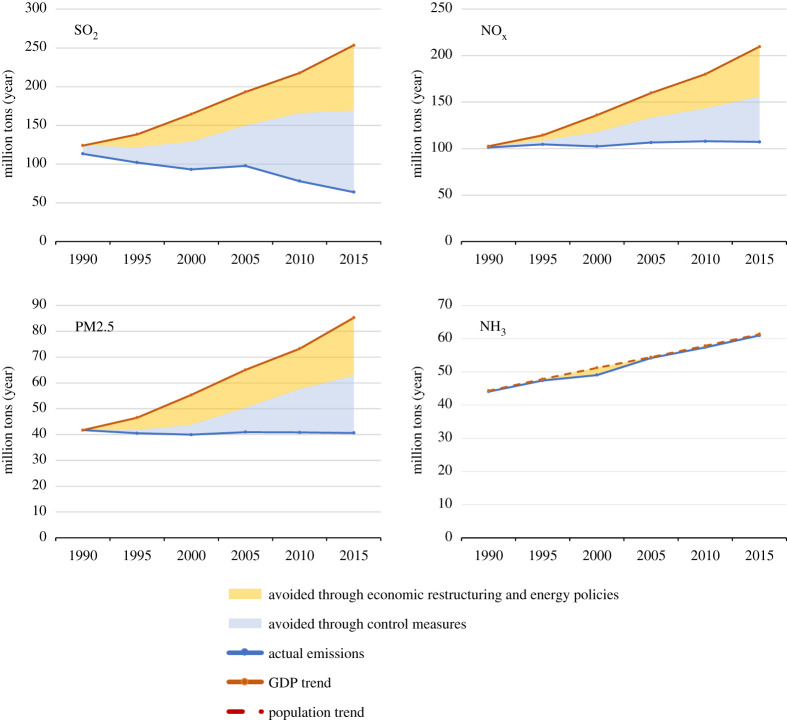
Factors contributing to the decoupling between the GDP trend and the evolution of global precursor emissions of PM2.5 from anthropogenic sources between 1990 and 2015 (source GAINS). (Online version in colour.)

For energy-related emissions, a global level decomposition analysis apportions about half of the past divergences of economic and pollution trends to environmental policies, while the remainder occurred from economic structural changes and energy policies (figure 3). By contrast, for global agricultural NH_3_ emissions no impacts from structural changes nor from pollution control policies can be identified. However, trends differ across world regions.

### (b) PM2.5 concentrations and population exposure in 2015

**Figure 4.**
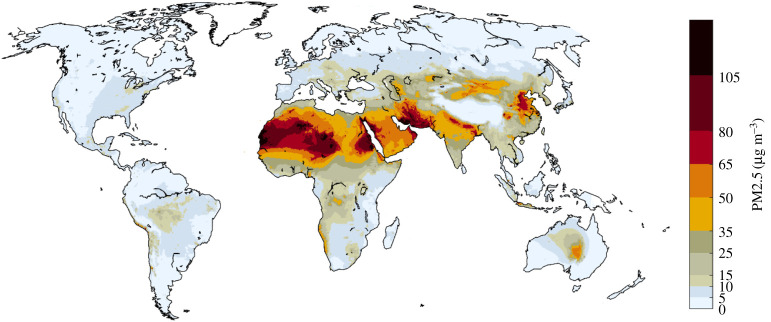
PM2.5 concentrations in 2015 as computed for this paper with the GAINS model, including contributions from natural sources. (Online version in colour.)

**Figure 5.**
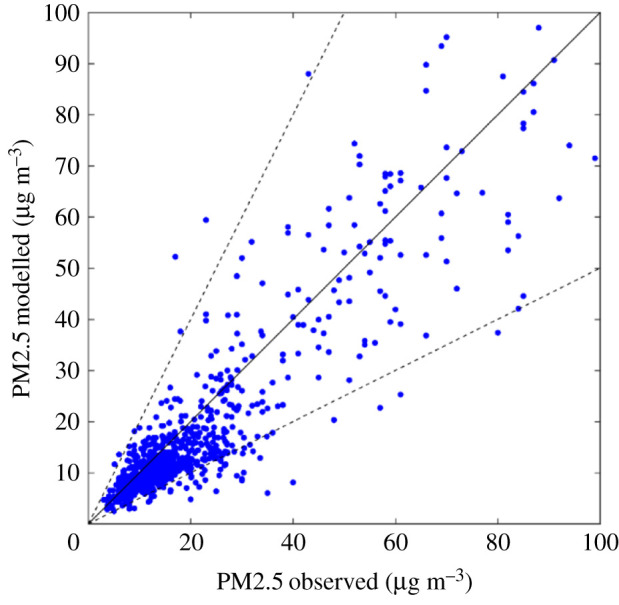
Comparison of monitoring data (annual mean concentrations in 2015) for PM2.5 with GAINS model results for 2015. Monitoring data taken from [54,55]. (Online version in colour.)

For 2015, calculations confirm large differences in PM2.5 concentrations across the world (figure 4), with patterns rather similar to other studies that have been derived from a limited set of ground-level monitoring data [1] or from interpretation of remote sensing products [52,53]. A comparison of ground-based monitoring data [54,55] with model calculations is presented in figure 5.

The main sources of PM2.5 in ambient air vary over world regions [56]. While in many densely populated or highly industrialized areas the major share originates from anthropogenic sources, natural sources (soil dust, sea salt and vegetation) are important in other areas, with soil dust dominating in arid regions in Asia, Africa, Latin America and Australia. Human activities might contribute only indirectly to these natural emissions, e.g. through desertification, land use changes and climate change. In general, such emissions do not appear to be controllable at the decadal time scale, as this would require geo-engineering interventions at the large scale.

**Figure 6.**
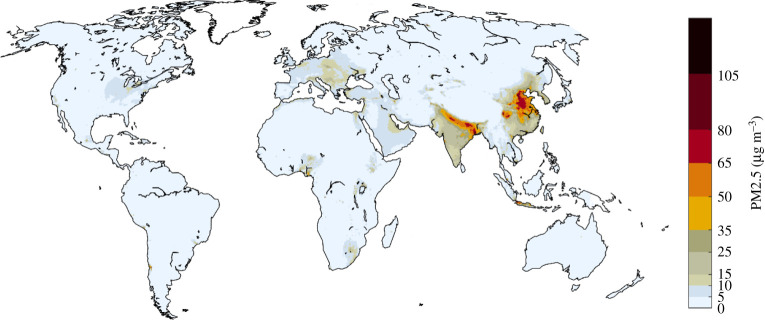
Computed PM2.5 concentrations in 2015 from anthropogenic emission sources only (contributions from natural sources are excluded). (Online version in colour.)

With a focus on future policy interventions, PM2.5 concentration fields that emerge from anthropogenic emission sources only are of special interest. For 2015, the highest hot spots caused by human activities are computed for eastern China and the Indo-Gangetic plain; concentrations exceeded 10 µg m^−^³ in large areas in China, Korea, Indonesia, throughout India, in central and eastern Europe, the Po valley and in the Benelux area, as well as in the Gulf States, the lower Nile valley, Nigeria, and around Johannesburg in South Africa (figure 6).

**Figure 7.**
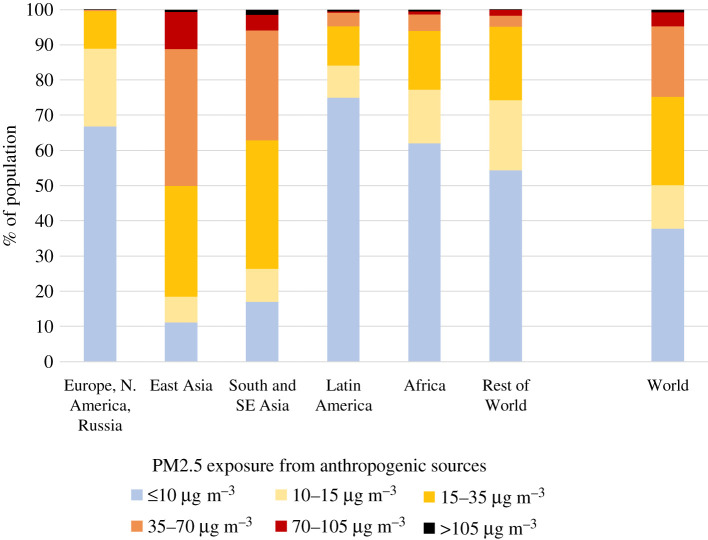
Distribution of population exposure to PM2.5 from anthropogenic sources in 2015. (Online version in colour.)

While there is little evidence to suggest a threshold below which no adverse health effects would be anticipated, the WHO has issued a global air quality guideline value for PM2.5 in ambient air of 10 µg m^−^³ as annual mean, together with a series of interim targets of 15, 25 and 35 µg m^−^³, respectively [57]. For 2015, it is estimated that globally more than 90% of the city dwellers [54] and more than 80% of the total population (this study) were exposed to PM2.5 levels above the WHO guideline, especially in Asia, Africa and the Middle East. Focusing on the exposure that can be influenced by policy decisions, more than 60% of the global population (85–90% in Asia) lived in areas where PM2.5 concentrations from anthropogenic sources exceeded 10 µg m^−^³ (figure 7).

### (c) Air quality futures

In the past, changes in air pollutant emissions have been driven by economic development and policy choices [13,19]. There is general agreement in the scientific literature that in the long run, i.e. in the second half of the twenty-first century, continuing growth in economic wealth, technological development and hypothesized further policy interventions should lead to significant declines in energy- and poverty-related emissions [24]. However, the prospects for the first half of the twenty-first century are less clear, especially when and at what level emissions are likely to peak in the various world regions. Although comparably less work has addressed future agricultural emissions [58], there are hardly any studies that would indicate a possible peaking, e.g. of NH_3_ emissions, at the global scale [59].

To shed light on the question of emission peaking and the role of policy interventions, this paper examines the prospects of air pollutant emissions, air quality and population exposure for a range of alternative policy assumptions. The analysis extends to 2040.

#### (i) Macro-economic trends

Although there is some uncertainty about future socio-economic development trends, the current scientific literature sees only moderate differences for the next few decades (e.g. until 2040) due to the long time scales of fundamental transformations of the social and economic systems. Thus, this paper adopts the macro-economic development assumptions of the World Energy Outlook 2018 of the International Energy Agency (IEA) [60], which up to 2040 are rather similar to the middle range of the long-term climate scenarios developed for the CMIP6 Coupled Model Intercomparison Project [28,61]. In total, the trends in 25 world regions of the IEA scenario correspond to a 25% growth in world population between 2015 and 2040. Global GDP increases by about 90%, so that global *per capita* income rises by 50%.

#### (ii) Policy scenarios

Three scenarios explore the potential impact of policy interventions on future air quality:

— As a benchmark for the quantification of the benefits from already implemented air pollution policies, a ‘Without Air Pollution Policies' scenario hypothesizes a case in which governments would not have adopted any dedicated air pollution controls beyond the basic technologies that are required for proper operations of plants (e.g. basic particulate matter removal technologies in industry and power plants). To facilitate direct comparability with the other policy scenarios, projections of emission-generating activities follow the ‘New Policies’ scenario of the IEA World Energy Outlook 2018 (see below).
— By contrast, a ‘2018 legislation’ scenario illustrates the implications of the emission-relevant policy interventions that were in place or have been issued by 2018. These include:
  1. Energy policies and measures that were already put in place in 2017 or announced in official targets and plans, as reflected by the ‘New Policies Scenario’ of the IEA World Energy Outlook 2018 [60]. *Inter alia*, the scenario includes the Nationally Determined Contributions (NDCs) of the Paris agreement, based on the IEA assessments of the relevant political, regulatory, market, infrastructure and financial constraints.
  2. Food and agricultural policies: Along the projection of the Food and Agriculture Organization (FAO) [62], this scenario extrapolates current consumption trends for the assumed population and income growth, combined with ongoing technological progress and changes in agricultural practices.
  3. National air pollution control policies and measures: The scenario assumes, for each of the 180 world regions, effective implementation of all air pollution measures that have been adopted as of 2018, according to the agreed time schedule. In particular, it includes the advanced emission controls that are currently required in industrialized countries, the current penetration of SO_2_, NO_x_ and PM controls of large point sources in many developing countries, and the latest national vehicle emission standards (see electronic supplementary material). For international shipping, the latest agreements of the International Maritime Organization (IMO) on the limitation of the sulfur content in marine fuels [63] are considered.
— A Clean Air scenario explores the theoretical potential of achieving clean air worldwide through a combination of further ambitious policy interventions in four areas, i.e. (i) traditional air pollution policies, (ii) energy and climate policies, (iii) agricultural policies and (iv) food policies. In particular, the scenario assumes full implementation of the best emission control technologies that are currently available on the market and, with a visionary perspective, policies and measures that are discussed in the context of societal transformation towards global long-term sustainability [64]. These include decarbonization strategies to achieve the Paris climate accord and keep global temperature increase well below 2°C, dietary changes to optimize human health and environmental sustainability, modifications of current agricultural practices to minimize alterations of the global nitrogen and phosphorus cycles, and other policies to achieve the UN Sustainable Development Goals, e.g. on waste management, circular economy, etc. The various measures are discussed in the scientific literature and are included in the scenario to the extent that they are conceived as technically feasible in the next few decades. Obviously, at present many of these measures find little political support, and their implementation will strongly depend on appropriate political will. In particular, the Clean Air scenario comprises four sectoral policy packages :
  (i) The Air Pollution Policy package assumes for all energy-related emission sources the most effective technical pollution control measures that are currently applied in the world. For large stationary combustion sources, measures include end-of-pipe control of SO_2_, NO_x_ and PM through, e.g., flue gas desulfurization, de-NO_x_ catalysts and electrostatic precipitators. Technological improvements, reduction of fugitive losses and end-of-pipe controls are also applied to industrial processes, while for small and often informal industries (e.g. brick production) shift to more efficient and less polluting technologies is assumed. For mobile sources, the scenario considers effective introduction of, and compliance with, Euro-6 equivalent emission standards for all new vehicles and machinery. For international maritime shipping, the scenario assumes global extension of the current regulations for the SO_x_ and NO_x_ Emission Control Areas (SECAs and NECAs) in the European seas, i.e. 0.1% sulfur content and selective catalytic reduction (SCR) to reduce NO_x_ for all new vessels. For the agricultural sector, it considers improved alternative manure management practices, i.e. covered storage and low emission application of manure to fields, as well as optimized application of synthetic fertilizers (especially of urea). In addition, this policy package includes a set of development measures to reduce highly polluting practices, including the open burning of municipal waste and agricultural residues, excessive fireworks, open-air kitchens, cremation.
  (ii) Accounting for physical and technical constraints to full implementation, the analysis in this paper assumes that—given adequate political will—introduction of such measures would begin at all new sources in 2020, without premature scrapping of existing capital stock before the end of its technical lifetime. Whereas further technological progress is likely to enhance removal efficiencies, extend their applicability to a wider range of emission sources and reduce costs, the analysis employs the current technical features, however, assuming that technologies are properly operated everywhere.
  (iii) The Energy and Climate Policy package includes policy interventions that are required for the decarbonization of the energy system. The scenario builds on the ‘Sustainable Development Scenario’ of the IEA World Energy Outlook 2018 that aims at the Sustainable Development Goals (SDGs) of the United Nations. It is aligned with the goal of the Paris agreement to hold the increase in the global average temperature to well below 2°C above pre-industrial levels. The scenario assumes that Sustainable Development Goal 7.1, achieving full access to electricity and access to clean cooking is met. It also assumes that countries implement a host of policies to reduce energy-related CO_2_ emissions. Those include policies to decarbonize the power sector by around 2050: sharp increase in solar and wind, increase in nuclear and, where economically viable, use of carbon capture and storage. This leads to a sharp decline in coal use. In transport, a combination of efficiency measures for conventional cars and trucks, electrification and fuel switch (to biofuels, natural gas and hydrogen in the longer term) are taken into account. By 2040 nearly half of the world's car fleet will be electric, which will lead to important reductions of exhaust emissions, while no major impacts are expected for emissions from road abrasion, tyre and break wear. Efficiency, electrification and fuel switching are also adopted in buildings and industrial sectors.
  (iv) Focusing on the agricultural sector, the Agricultural Policy package considers massive modifications of agricultural practices to reduce NH_3_ and greenhouse gas emissions to the atmosphere. Industrial farms would introduce enclosed systems for manure treatment (e.g. anaerobic digestion, composting), whose residual products would be used as soil amendments to replace mineral fertilizers. Non-industrial/small farms would either deliver manure to central processing facilities or extend grazing of animals. Furthermore, to reduce nitrogen losses, policies would aim to increase nitrogen use efficiency by optimizing nutrient additions to crop requirements and by minimizing excess protein in animal feed, to reach the maximum nitrogen use values supported in the scientific literature [58]. Also, well-managed and healthy animal stocks that are genetically well adapted to the local environment could simultaneously enhance productivity, fertility and longevity. This would allow a reduction in the number of non-productive animals in the stock, and thereby minimize emissions per unit of meat or milk produced [65].
  (v) The Food Policy package aims to reduce emissions from meat production through modified human diets and lower food waste. Policies would promote the ‘Planetary Health Diet’ proposed by the EAT-Lancet Commission on Food, Planet and Health to address the simultaneous global problems of malnutrition (under-nutrition) and over-nutrition [66]. The shift from animal-based protein to plant-based protein, together with reduced food waste [67], would decrease meat consumption and allow smaller herd sizes and less mineral fertilizer use for animal feed production. The scenario employs the quantitative implications on future livestock numbers and mineral fertilizer use developed with the GLOBIOM model [68] for the Food and Land Use Coalition [69].

#### (iii) Future emissions

**Figure 8.**
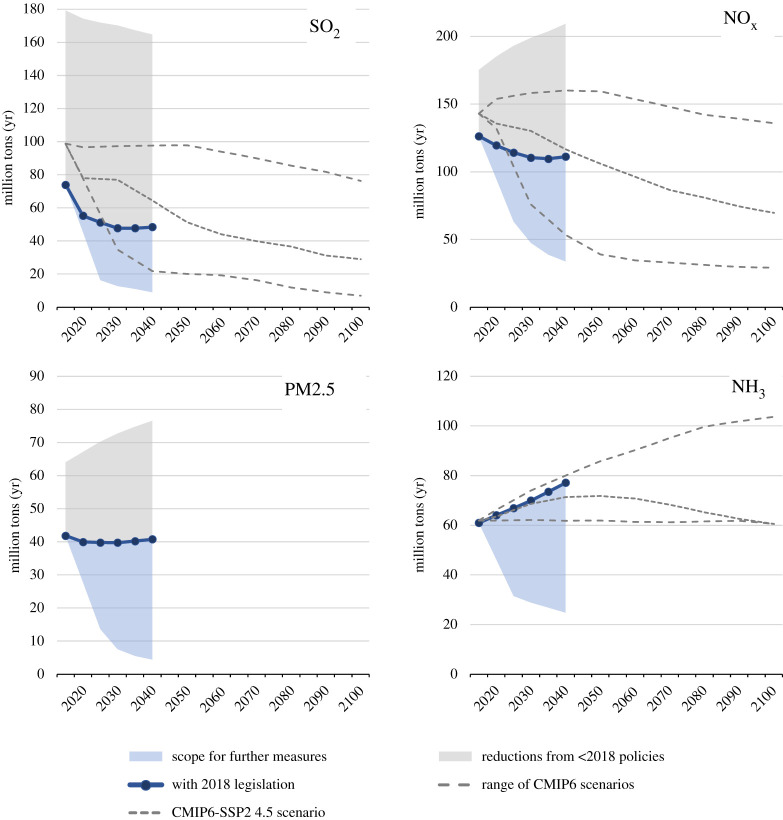
The range of future global emissions (2015–2100) resulting from alternative assumptions on policy interventions. The grey area indicates the emission reductions from the policies and measures that have been adopted and implemented until 2018, while the blue areas outline the scope for additional policy interventions. The dark blue line indicates the ‘2018 legislation’ scenario which assumes that all air pollution controls that have been decided by 2018 will be timely and effectively implemented and enforced. For comparison, the dashed grey lines illustrate the range of the long-term emission scenarios used for the CMIP6 climate model calculations. Note that CMIP6 does not provide PM2.5 emissions. (Online version in colour.)

The different assumptions on policy interventions deliver a wide range of future emission trends for PM2.5 precursors (figure 8; electronic supplementary material). By 2040, without policies and measures introduced in the last decades, energy-related emissions could have been up to 120% higher than in 2015. Current policies, if effectively implemented and enforced, would reduce global SO_2_ emissions by about one-third and NO_x_ by about 10%, and stabilize global PM2.5 emissions. By contrast, with adequate political will, global PM2.5 and SO_2_ could be cut by about 90% below today's level, and NO_x_ by about 70%. However, current policies will do little to decouple global NH_3_ emission trends from population growth, while ambitious interventions have a potential to enable 60% lower emissions. However, as this analysis does not consider potential impacts of climate change on meteorological conditions, changes in emissions from natural sources, e.g. desert dust and biogenic VOCs, are not excluded from the analysis. These could potentially enhance wind-blown PM2.5 and enhance ground-level ozone.

**Figure 9.**
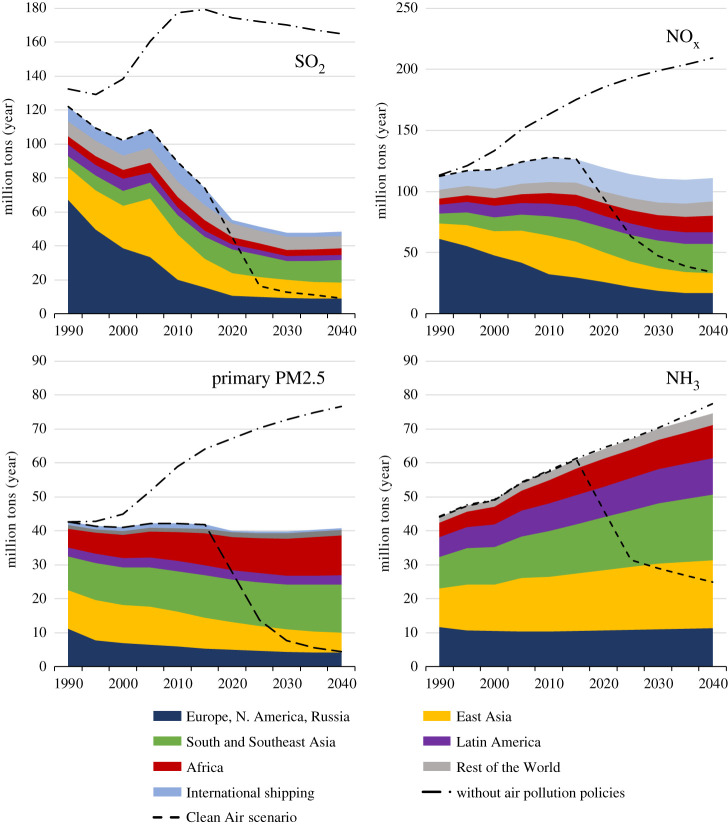
Emission trends 1990–2040 assuming effective implementation and enforcement of all pollution controls that were decided by 2018 (Scenario: 2018 legislation). For comparison, the global trends for the ‘Without air pollution policies' and the ‘Clean Air’ scenarios are provided. (Online version in colour.)

In general, the range of future emissions developed for this paper is consistent with the scenarios of the CMIP6 Coupled Model Intercomparison Project [28,61,70] of the World Climate Research Program. However, the ‘2018 legislation’ scenario indicates stabilizing or even rebounding emissions towards 2040 after full implementation of current policies, while emission declines prevail in CMIP6. Also, the global trend masks diverging developments in some world regions. Especially, in many Asian and African countries, the current pollution control policies seem insufficient to counteract pressure on NO_x_, PM2.5 and NH_3_ emissions from economic growth (figure 9).

#### (iv) Future air quality and population exposure

**Figure 10.**
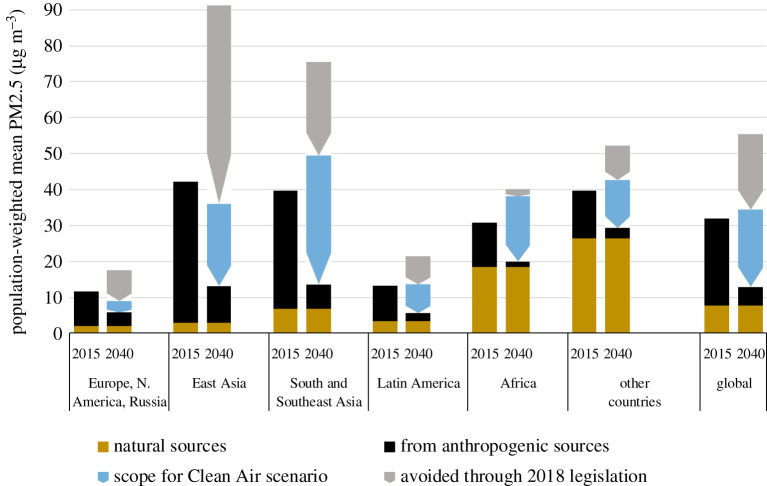
Reductions in population-weighted mean exposure to PM2.5 in the six world regions. (Online version in colour.)

Changes in emissions, prominently determined by policy decisions, will impact future air quality and population exposure. By 2040, effective implementation of the 2018 legislation would bring PM2.5 concentrations from anthropogenic sources below their respective 2015 levels in Europe, North America, and North and East Asia. In other regions, concentrations would grow further, notably in South Asia and Africa (+25%). Global population-weighted annual mean concentrations would increase by 10% to about 35 µg m^−^³ (including contributions from natural sources) (figure 10). At the same time, due to the improvements in industrialized countries, the share of the global population living in areas where PM2.5 complies with the current WHO guideline would increase to 23% compared to 18% in 2015.

**Figure 11.**
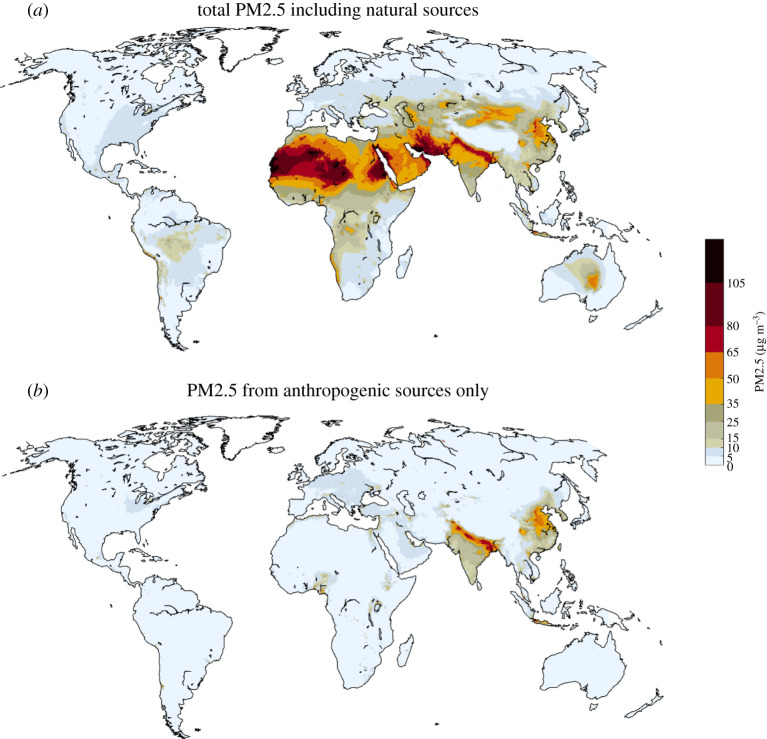
Modelled PM2.5 concentrations in 2040 for the 2018 legislation case. Upper panel: total PM2.5 including anthropogenic and natural sources; lower panel: contributions from anthropogenic sources only. (Online version in colour.)

Highest PM2.5 concentrations would then occur in Africa and the Middle East, where concentrations would reach the levels currently prevailing in Asia (figure 11*a*). Anthropogenic sources would make the largest contributions in eastern China and the Indo-Gangetic plain and increase by 60% in Africa and 30% in the Middle East (figure 11*b*).

**Figure 12.**
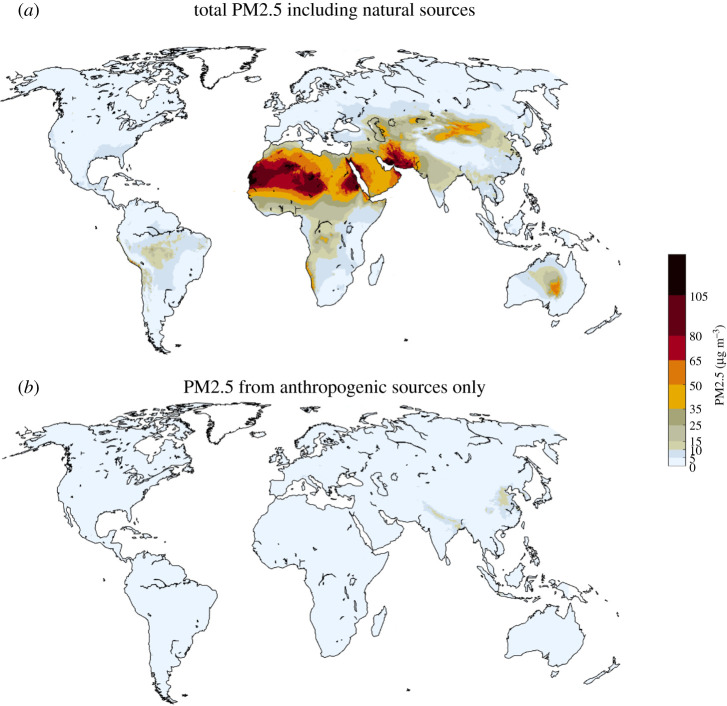
Modelled PM2.5 concentrations in 2040 for the Clean Air scenario. A: total PM2.5 including anthropogenic sources; B: contributions from anthropogenic sources only. (Online version in colour.)

By contrast, the ambitious policies and measures of the Clean Air scenario would deliver significant cuts in PM2.5 levels throughout the world. In Europe and the Americas, population-weighted concentrations could be brought down to about 5 µg m^−^³ (including natural sources), in Asia to about 15 µg m^−^³ and in Africa and Middle East to 20 and 30 µg m^−^³, respectively (figure 12*a*). Fifty-six per cent of the global population would then live in areas where PM2.5 complies with the current WHO guideline.

Natural sources will continue to make sizeable contributions to population exposure, especially in Africa, Latin America and the Middle East. In these regions, the Clean Air scenario would bring contributions from anthropogenic sources to below 3 µg m^−^³ (figure 12*b*).

**Figure 13.**
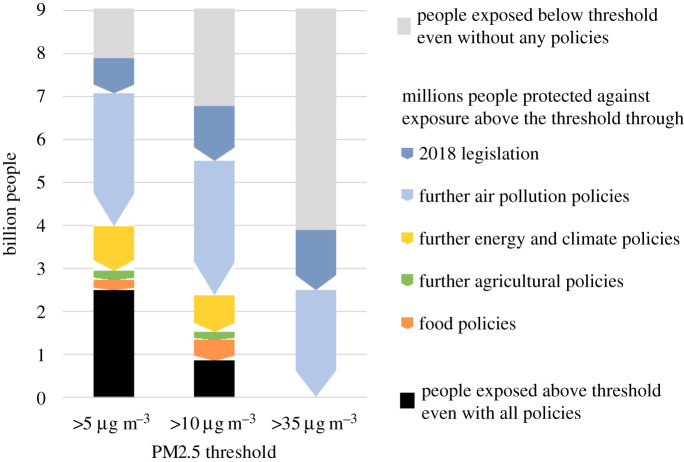
Scope of the policy packages to protect people from PM2.5 exposure from anthropogenic sources in 2040. (Online version in colour.)

The significant exposure reductions of the visionary Clean Air scenario emerge from a range of policies and measures that have been grouped into four policy packages: (i) environmental pollution control policies, (ii) energy and climate policies, (iii) agricultural policies, and (iv) food policies (figure 13). Further environmental pollution controls offer the largest improvements in population exposure. However, even full implementation according to the assumptions in this paper would still leave about 2.4 billion people exposed to more than 10 µg m^−^³ PM2.5 from anthropogenic sources. Beyond this, energy and climate measures could reduce exposure below this level for another 820 million people, changes in agricultural practices for 670 million people and the assumed food policies for an additional 500 million people. Thus, further air pollution control policies would exhaust only 60% of the total potential offered by the full range of policy interventions. Vice versa, the notion that other sectoral policies such as ambitious energy and climate policies would resolve air pollution problems on their own, e.g. [23], is not supported by this analysis, and a clear role for dedicated environmental air quality policies will remain in the future.

### (d) Co-control of emissions of greenhouse gases and short-lived climate pollutants

**Figure 14.**
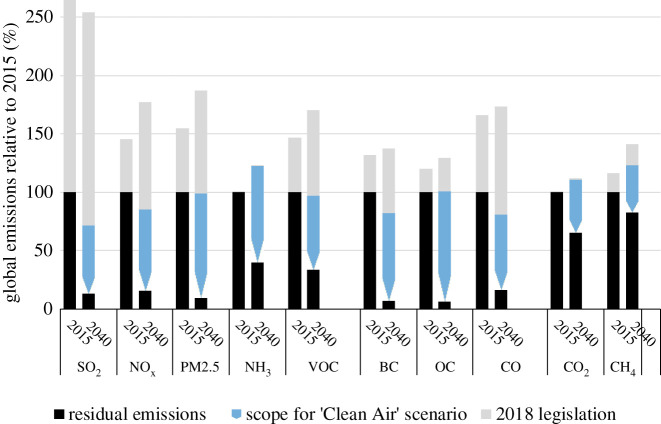
Emission reductions from the policy scenarios. 2018 legislation includes all controls that have been decided by 2018. (Online version in colour.)

Some of the measures of the Clean Air scenario will not only reduce emissions of PM2.5 precursors but simultaneously also other substances that contribute to temperature increase. As a consequence of the deep energy system restructuring in the Clean Air scenario, CO_2_ emissions in this scenario in 2040 are 40% lower than in the 2018 legislation reference case, CH_4_ 33% and black carbon 90% lower (figure 14). Quantification of the temperature impact of these cuts in long-lived greenhouse gases and short-lived climate pollutants is beyond the scope of this paper. We also did not quantify reductions of N_2_O emissions, which can be expected as a result of reduced application of mineral fertilizer and more efficient use of manure nutrients.

As this paper focuses on health impacts from PM2.5, the Clean Air scenario does not target substances that contribute to other air pollution problems. SO_2_, NO_x_ and NH_3_ emissions, which affect acidification and eutrophication of ecosystems, are included as PM precursors. For ground-level ozone, NO_x_ and VOC emissions are considered as PM precursors as well. However, CH_4_ and CO emissions are not addressed here, as their contribution to PM levels is considered marginal. A comprehensive clean air policy that would also aim at ground-level ozone would need to include CH_4_ and CO mitigation in its portfolio, especially in view of the past increases in hemispheric ozone levels that have been attributed to growing CH_4_ emissions [71]. From such a perspective, measures that reduce only CH_4_ but would not affect PM precursor emissions could cut global CH_4_ by more than half in 2040 [72].

### (e) Co-benefits on the sustainable development goals

The Clean Air scenario achieves a substantial reduction in exposure to PM2.5, bringing about significant progress in moving towards the WHO air quality guideline value for PM2.5, which is currently set at 10 µg m^−^³ including contributions from natural sources. In large areas of the world, the Clean Air scenario would reduce anthropogenic PM2.5 below 5 µg m^−^³ and thereby leave space for contributions from natural sources. These improvements will directly contribute to SDG 3 (Improve human health and wellbeing), particularly in urban areas (SDG 11—sustainable cities and communities).

In addition, the measures assumed in the Clean Air scenario with the focus on health impacts from PM would deliver a host of co-benefits in other policy areas through several pathways.

— The measures would lead to considerably lower emissions of greenhouse gases (CO_2_, CH_4_) and black carbon, which will reduce temperature increase and thereby contribute to SDG 13 (Climate action).
— The policies and measures in the energy sector will enhance energy efficiency and provide access to clean energy (SDG 7—Affordable and clean energy). In addition, enhanced access to clean household fuels will eliminate the health impacts from indoor exposure to PM from household use of traditional solid fuels, currently estimated to cause another three million cases of premature deaths annually [73].
— As an important feature, the Clean Air scenario contains a host of measures that will enhance nitrogen use efficiency. This will secure crop production (SDG 2—Zero hunger; SDG 12—Responsible consumption and production) and deliver positive impacts on the global nitrogen and phosphorus cycles. Nitrogen runoff to water (SDG 14—Life under water) will be reduced and protect biodiversity through reduced ecosystems exposure to excess nitrogen deposition (SDG 15—Life on land).

## 4. Discussion and conclusion

### (a) Discussion

This paper develops an initial global vision on policy interventions that could bring PM2.5 levels close to or below the WHO air quality guideline value in the next few decades. The analysis takes a holistic perspective, connecting past trends of economic and technological development, atmospheric dispersion and the role of policy interventions with a focus on improving human wellbeing through better global air quality.

The wide range of aspects included in the analysis and the pioneering character of this paper leave a number of issues that deserve further attention.

First, the paper focuses on population exposure to PM2.5 in ambient air, as associated health impacts have been found to dominate other negative consequences of air pollution in economic terms [74–76]. Thereby, the paper does not explicitly address health impacts from ground-level ozone [77], vegetation damage from ozone including economically important losses in crop production [78,79], biodiversity impacts of excess nitrogen deposition [80] and acid deposition that exceeds the absorption capacity of ecosystems [81].

For the later issues, i.e. excess nitrogen and acid deposition, the Clean Air scenario delivers significant reductions in precursor emissions as these contribute to PM2.5 formation too. By 2040, NO_x_ would be lower by three-quarters, NH_3_ by two-thirds and SO_2_ by 80% than compared to the 2018 legislation projection. These reductions will alleviate pressure on ecosystems, but the benefits have not been quantified for this paper.

Similarly, the Clean Air scenario, even while aiming at ambient PM2.5, would lead to much lower precursor emissions of ground-level ozone. Global NO_x_ emissions would decline by about three-quarters and global VOC emissions by about two-thirds compared to the 2018 legislation case, because of their role in the formation of secondary particles. In addition, the measures would also reduce the other precursors of ground-level ozone that are often neglected in local and regional pollution control strategies. Global CH_4_ emissions, which have an important impact on hemispheric background levels of ozone [82], would decline by 33% as a side-effect of the changes in fossil fuel production, agricultural practices and food demand that are motivated by the interest to reduce PM precursor emissions including NH_3_. More work will be required to quantify the consequences of these emission changes on ozone fields and resulting health and vegetation impacts.

Second, the paper limits the analysis to 2040, in order to explore near-term policy interventions without excessive speculation about long-term technological, socio-economic and climate trends. At the same time, the long-term transformational energy and agricultural policies that are assumed in the Clean Air scenario involve measures that will fully unfold only by 2050, so that the 2040 focus does not reveal their full potential in the long run. Additional work would be required to assess the impacts beyond 2040.

Furthermore, any visionary analysis requires assumptions and methods that are associated with uncertainties. The following aspects have been identified as particularly relevant.

— **Political will:** The Clean Air scenario deliberately assumes a range of ambitious policy interventions that clearly deviate from current practices. Implementation will require clear political will and public support. It is the purpose of this paper to reveal the multiple benefits from such actions and thereby contribute to enhanced societal push for such policies.
— **Technical feasibility of measures:** Care has been taken to compile a portfolio of technical measures that is likely to be feasible from a technical perspective within the coming decades, given appropriate political will. While some of the measures appear as visionary, they are discussed in other contexts as necessary for the transformational changes that are required for achieving global sustainability. The emphasis in this paper is to reveal the co-benefits of such measures on clean air that are often overlooked, and to explore how they could contribute to clean air policies, in addition to their core policy objectives.
— **Importance of emission sources that receive less attention today:** Assuming the drastic reductions in PM2.5 precursors from the Clean Air scenario, other sources which make only marginal contributions today will dominate remaining emissions. These include, *inter alia*, tobacco smoking, emissions from barbecues and food preparation, fireworks, solvents embedded in cleaning agents and cosmetics, etc. While in theory mitigation measures are conceivable for such sources, limited experience with interventions other than changing human behaviour exists up to now.
— **Climate change impacts on biogenic emissions:** Calculations include emissions from vegetation, e.g. biogenic emissions of volatile organic compounds, based on current estimates. However, a host of the literature indicates potential changes/increases of such emissions from climate and land use changes [83] which are not considered in this analysis.
— **Uncertainties inherent in future development**: The paper adopts a ‘middle of the road’ projection of socio-economic development up to 2040, for which, however, alternative projections show only limited differences. Implications of alternative development paths need to be further assessed, especially for longer time horizons.
— **Atmospheric dispersion calculations**:
  1. **Spatial resolution:** Calculations are conducted at a spatial resolution of 0.1° × 0.1° at the global scale and even higher resolution in Europe, which produces a great deal of spatial detail. However, uncertainties remain about the quality of input data at this fine resolution (e.g. emission inventories for all pollutants, spatial distribution of emissions, meteorological data, topographic features, etc.) and their anticipated development over time for different socio-economic scenarios.
  2. **Use of a reduced-form model:** The calculations of atmospheric chemistry and transport of pollutants employ a reduced-form model that has been derived statistically from a large set of model simulations produced with a state-of-the-art Eulerian chemical transport model. The validity of the statistical model for the deep emission cuts of the Clean Air scenario has been tested and validated with the full CTM, indicating a slightly conservative bias of the statistical model (PM2.5 concentration changes are underestimated by typically not more than 1 µg m^−^³, even in areas with high air pollution).
  3. **Constant meteorological conditions:** Calculations of ambient PM2.5 changes under different emission scenarios are done with constant observed meteorological conditions (2009 in Europe, 2015 for the rest of the world). While this approach makes the effects of PM2.5 precursor emission changes comparable between scenarios, it ignores possible changes in dispersion patterns under a changed future climate. Furthermore, the specific conditions in any given year may deviate from these in our base year, leading to higher or lower exposure.
  4. **Quantification of emissions from natural sources:** There is only imperfect understanding of the source strengths of natural emission sources (e.g. soil dust, sea salt, biogenic emissions) and temporal patterns and their distribution at the global scale. The approach chosen for this paper, i.e. to focus on the anthropogenic fraction of PM2.5, attempts to minimize the impact of these uncertainties on the findings. However, uncertainties are particularly relevant when estimating population exposed to specific PM2.5 concentrations above or below a given threshold, e.g. the WHO guideline value which includes natural sources. Especially at low concentrations, differences in the contributions from natural sources of a few µg m^−3^ could easily double the number of people that are exposed to such levels.
— **Other air pollutants:** With a focus on human health and following epidemiological evidence, this paper addresses exposure to fine particulate matter (PM2.5) as the pollutant causing the majority of health impacts. Obviously, health impacts have been identified from exposure to other substances as well, notably to ground-level ozone. However, as all quantifications of health impacts from air pollution reveal a dominating role of PM2.5 over all other substances [48], this initial analysis has been restricted to PM2.5. It would be important to extend the analysis to ground-level ozone in the future. However, the key precursor emissions of ground-level ozone, i.e. NO_x_ and VOC, are considered as precursors of PM2.5 in the atmospheric chemistry calculations of this paper as well (results for VOC are shown in the electronic supplementary material).
— **Quantification of health benefits:** Although a host of literature has produced a large array of estimates of the health impacts of air pollution at the global scale [1,74,84,85], uncertainties remain, *inter alia*, about the general shape of the exposure-response functions [86], the levels of counterfactual concentrations, and the validity of the epidemiological evidence at the global scale. In particular, current methods applied for global analyses include PM exposure from natural sources in the assessment, while for European assessments WHO Europe recommended a focus on anthropogenic sources [87]. Given new epidemiological evidence, *inter alia* on the relevance of health impacts at low concentrations [88], the World Health Organization is currently reviewing and possibly revising the current air quality guidelines. In order not to pre-empt the results of that review, this paper refrains from the quantification of health impacts and restricts the assessment to population exposure.
— **Economic aspects:** The paper does not address the cost-effectiveness and economic feasibility of the policies and measures. Also, the Clean Air scenario assumes (ambitious) measures throughout the world, resulting in many world regions in rather low PM2.5 concentrations. To the extent that higher concentrations appear acceptable, a cost-effectiveness analysis could reveal a sub-set of measures that deliver benefits at lowest cost.

### (b) Summary

Over the last decades, emissions of key air pollutants have decoupled from economic growth at the global level, and increasingly also in the developing world. This trend break was to a large extent a result of policy decisions on pollution control and energy, in addition to structural changes in the economy and in consumption patterns. It is estimated that without dedicated pollution control policies, global SO_2_ emissions would have been more than twice as high as in 2015, and PM2.5 and NO_x_ emissions twice as high. Given the lack of large-scale policy interventions, the evolution of agricultural NH_3_ emissions closely followed global population growth.

The dominating role of policy interventions will prevail in the future. Instead of autonomous improvement of air quality connected with increasing economic wealth as hypothesized in the environmental Kuznets curve, this analysis reveals that future air quality will be mainly determined by policy decisions and their implementation. Timely implementation and full compliance with all air legislation that has been decided by 2018 is likely to decrease global anthropogenic SO_2_ emissions by 35% by 2040, and NO_x_ and primary PM2.5 by about 10%. By contrast, for NH_3_ emissions a growth of one-quarter is estimated, with no peaking in sight. However, as illustrated by the Clean Air scenario, with adequate political will further policy interventions could cut global SO_2_ and PM2.5 by about 90%, NO_x_ by 70% and NH_3_ by 60% below today's levels. Decisions in four policy areas will be critical: environmental policies focusing on pollution controls, energy and climate policies aimed at global temperature stabilization in line with the Paris Agreement, policies to transform the agricultural production system, and policies to shift human food consumption patterns towards largely plant-based diets such as the ‘Planetary Health Diet’ proposed by the EAT-Lancet Commission on Food, Planet and Health. However, emissions from biogenic sources (e.g. VOC) might further increase due to other factors, e.g. climate change.

Obviously, the policy interventions considered in the Clean Air scenario would require fundamental transformations of today's practices in many sectors. These are visionary but considered likely to be technically achievable in the future. As they exceed current policy ambitions, their implementation would require strong political will.

Political will could emerge from a solid understanding of the full range of benefits. Most importantly, the policies and measures of the Clean Air scenario would drastically improve air quality throughout the world. Population exposure to PM_2.5_ from anthropogenic sources would decline by about 75% relative to 2015, or by 80% compared to a future without additional policies. This would deliver substantial health benefits and avoid a large portion of the 3–9 million cases of premature deaths from ambient air pollution that are estimated for 2015. In addition, enhanced access to clean household fuels will drastically reduce the health impacts from indoor exposure to PM2.5, currently estimated to cause another 1.6–4 million cases of premature deaths annually [3,73,89].

These reductions in PM2.5 exposure from anthropogenic sources would allow substantial progress in moving towards the WHO air quality guideline value, which is currently set at 10 µg m^−^³. In large areas of the world, the Clean Air scenario would reduce anthropogenic PM2.5 concentrations below 5 µg m^−^³ and thereby bring total PM2.5 below the current WHO guideline value, which includes contributions from natural sources. However, violations will remain in areas where natural sources alone deliver already more than 10 µg m^−^³, as well as at small-scale hot spots which are not captured by the spatial resolution of this analysis.

In addition to improved human health (SDG 3—Improve human health and wellbeing), particularly in urban areas (SDG 11—Sustainable cities and communities), the Clean Air scenario would deliver a host of co-benefits in other policy areas through several pathways.

— For mitigating climate change (SDG 13—climate action), some of the measures will not only reduce emissions of PM2.5 precursors but will simultaneously reduce emissions that contribute to temperature increase. In particular, CO_2_ emissions of the Clean Air scenario will be about 40% lower than in the reference case in 2040, CH_4_ 33%, and black carbon by 90%.
— The policies and measures in the energy sector will enhance energy efficiency and provide access to clean energy (SDG 7—Affordable and clean energy).
— As an important feature, the Clean Air scenario contains a large number of measures to enhance nitrogen use efficiency, which will have positive impact on the global nitrogen and phosphorus cycles. This will contribute, *inter alia*, to SDG 2—Zero hunger, SDG 14—Life under water, SDG 15—Life on land, SDG 12—Responsible consumption and production, and SDG 13—Climate action.

Importantly, the Clean Air scenario includes a range of fundamental transformative changes (e.g. in the energy, agricultural and food systems) which are required for global sustainability and whose benefits occur globally and in the long term. The tangible local and near-term health benefits of the Clean Air scenario could enhance social acceptance and political support for such transformative policies.

Despite the importance of a deep involvement of and interactions with numerous other policy areas, the analysis reinforces the continued relevance of environmental air quality policies. Ambitious action in other policy domains such energy, climate, food and agriculture will not resolve the air pollution problem on its own and—if not coordinated within a comprehensive multi-sectoral air quality management approach—could even lead to counterproductive impacts on air quality.

### (c) Key messages

— Policy interventions were instrumental in decoupling energy-related air pollution from economic growth in the past, and further interventions will determine future air quality.
— At the global scale, even full implementation and enforcement of current policies are unlikely to reduce present exposure [and health burden] from air pollution in the next 20 years. Improvements in North America, Europe and East Asia will be compensated by further deterioration in South Asia, Africa and the Middle East.
— Theoretically, a portfolio of ambitious policy interventions could bring ambient PM2.5 concentrations below the WHO air quality guideline in most parts of the world, except in areas where natural sources (e.g. soil dust) contribute major shares to or even exceed the guideline value.
— Such a portfolio needs to involve (i) environmental policies focusing on pollution controls, (ii) energy and climate policies, (iii) policies to transform the agricultural production system, and (iv) policies to modify human food consumption patterns. None of these policy areas alone can deliver clean air, and interventions need to be coordinated across sectors.
— These policy interventions would require fundamental transformations of today's practices in many sectors. They are visionary but considered likely to be technically achievable in the future. As they exceed current policy ambitions, their implementation would require strong political will.
— Political will could emerge from a solid understanding of the full range of benefits. The policy package would avoid annually millions of premature deaths worldwide through drastic improvements in air quality and through healthier diets. It would reduce emissions that contribute to global temperature increase, alleviate distortions of the global nitrogen and phosphorous cycles, and enhance the protection of ecosystems and biodiversity. At the same time, policies would contribute to multiple UN Sustainable Development Goals and trigger transformations that are required for global long-term sustainability.
— Lowering emissions from agricultural activities will be critical for achieving clean air worldwide. The scientific understanding of the relevance of nitrogen emission reductions that has emerged over the last decade has not yet penetrated decision-making in many parts of the world, and possible measures often face strong resistance from interest groups. However, a focus on industrialized agriculture would provide competitive advantages to small and subsistence farmers. Also, some of the more advanced measures considered in the agricultural policy package reduce the greenhouse gas footprint of the agricultural sector as well.

## Supplementary Material

Amann et al., Controlling global air pollution: Development, economics and the scope for policy interventions in the 21st century

## Data accessibility

Data are accessible in the online version of IIASA's Greenhouse gas—Air pollution Interactions and Synergies (GAINS) model (http://gains.iiasa.ac.at). Data will be made available after acceptance of the paper. Key data are provided in the electronic supplementary material.
