# Supplementary material for: Reducing global air pollution: the scope for further policy interventions
Source: Philos Trans A Math Phys Eng Sci. 2020 Sep 28;378(2183):20190331. doi: 10.1098/rsta.2019.0331 (PMC7536039; doi:10.1098/rsta.2019.0331)
Supplement: Amann et al., Controlling global air pollution: Development, economics and the scope for policy interventions in the 21st century [file rsta20190331supp1.docx]

# Supplementary Information to Amann et al., Controlling global air pollution: Development, economics and the scope for policy interventions in the 21st century

## S.1. The GAINS model - Overview

The GAINS (Greenhouse gas-Air Pollution Interactions and Synergies) model explores cost-effective multi-pollutant emission control strategies that meet environmental objectives on air quality impacts (on human health and ecosystems) and greenhouse gases. GAINS, developed by the International Institute for Applied Systems Analysis (IIASA), brings together data on economic development, the structure, control potential and costs of emission sources, the formation and dispersion of pollutants in the atmosphere and an assessment of environmental impacts of pollution (http://gains.iiasa.ac.at).

GAINS has been used to address air pollution impacts on human health from fine particulate matter and ground-level ozone, vegetation damage caused by ground-level ozone, the acidification of terrestrial and aquatic ecosystems and excess nitrogen deposition to soils, in addition to the mitigation of greenhouse gas emissions. GAINS describes the interrelations between these multiple effects and the pollutants (SO_2_, NOx, PM, NMVOC, NH3, CO_2_, CH_4_, N_2_O, F-gases) that contribute to these effects at the regional scale.

GAINS explores, for each of the source regions considered in the model, the cost-effectiveness of more than 2000 measures to control emissions to the atmosphere. It computes the atmospheric dispersion of pollutants and analyses the costs and environmental impacts of pollution control strategies. In its optimization mode, GAINS identifies the least-cost balance of emission control measures across pollutants, economic sectors and countries that meet user-specified air quality and climate targets.

The global version of the GAINS model which is used for this study employs a spatially disaggregated representation of the world in 180 source regions, which are

- Countries: in Europe, South America, most of Asia, Canada, Mexico, Egypt, Nigeria, Tanzania, South Africa, Australia, New Zealand
- Provinces or sub-national aggregates: in China (35 provinces), India (23 states and aggregates), USA (Alaska and rest), several Asian countries
- Country aggregates: Carribean, Central America, 4 African regions

Activity projections are supplied by IEA’s World Energy Model (WEM) (IEA 2019) in the WEM native region, sector and fuel disaggregation. They are translated into the GAINS region, sector and fuel classification using the proportional downscaling algorithm reported by (Rafaj et al. 2013, 2018). The WEM model provides information on the future evolution of the energy system under various climate and energy policies for the following subsectors: power generation, fuel extraction and conversion, industry, transport and buildings. Not only combustion-related activities are modelled in WEM, also projections for industrial processes, e.g., iron and steel production, cement and aluminum manufacturing are developed. If some of the emission sources are not explicitly represented in WEM, they are derived from the socio-economic drivers such as population and economic growth, sectoral value added trends, etc. Examples of emitting sectors in GAINS not covered explicitly by WEM include livestock numbers, burning of agricultural residues, waste generation, brick production and other industrial process activities.

Energy consumption data from the WEM projections is distributed across the GAINS sub-regions (countries, states, provinces) based on shares derived from international and national energy and industrial statistics (see examples by (IEA 2020) or (Purohit et al. 2010),(Amann et al. 2017; Bhanarkar et al. 2018)(Cofala et al. 2015). The downscaling procedure also allocates energy consumption to detailed subsectors and fuel types in GAINS that are not explicitly provided by the energy model. These include various transport sub-categories, industrial demand activities split into furnaces/boilers as well as fuel conversion and processing.

For each of the source regions considered in GAINS, emission estimates for a particular emission control scenario consider (1) the detailed sectoral structure of the emission sources that emerges from the downscaling of the activity projection described above, (2) their technical features (e.g., fuel quality, plant types, etc.), and (3) applied emission controls (GAINS includes a database of over 1000 technical measures).

For each key source sector, the spatial patterns of PM and its precursors emissions are then estimated at a 0.5⁰ × 0.5⁰ longitude–latitude resolution, based on relevant proxy variables (updated from Klimont et al. 2017). These estimates rely on the most recent updates of data on population distribution, road networks, plant locations, open biomass burning, etc. that were originally developed within the Global Energy Assessment project (GEA 2012).

For the residential sector, a finer resolved emission distribution map has been developed at 0.1⁰ resolution, combining fine resolved gridded population with urban-rural classification, and estimates of prevalence of different fuel use in urban and rural areas.

Natural emissions are based on the estimates employed by the EMEP atmospheric chemistry and transport model (Simpson et al. 2012a).

# S.2 Ambient PM_2.5_ calculations in GAINS

The general principle of ambient PM_2.5_ calculations in GAINS has been discussed by (Amann et al. 2011). Owing to the history and evolution of the GAINS model over time, slightly different versions have been implemented in the European domain and in the global domain outside Europe. All versions rely on perturbation simulations of atmospheric CTMs, in which emissions from a given source region and pollutant are reduced from base case, and the change in ambient concentration levels is used to calculate a linear transfer coefficient. Source pollutants considered for the formation of PM_2.5_ are primary PM_2.5_ (PPM), SO_2_, NO_x_, NH_3_, and VOC. For PPM, the transfer coefficients are split into one describing low-level emissions from residential combustion and traffic, and one for all other sources, to account for different atmospheric dispersion characteristics of emissions injected at different heights.

Ambient PM_2.5_ calculations for Europe have been described by (Kiesewetter et al. 2015a, b). Linear transfer coefficients were derived based on EMEP model simulations (5 met years 2006-10) from region-pollutant specific emissions to 0.5⁰ x 0.25⁰ grid (Simpson et al. 2012b). They are then downscaled for low-level PPM within the grid cell to a finer 0.125⁰ x 0.0625⁰ grid (“7km”) and urban polygons inside the 7km grid, using a linear relationship between sub-grid PPM emission density and calculated PM_2.5_ concentrations derived from a full-year simulation of the CHIMERE CTM (Menut et al. 2013).

Low-level emissions considered for the downscaling include the domestic (SNAP 2), road transport (SNAP 7), and off-road transport (SNAP 8) sectors. Urban-rural split of emissions is done at the level of sub-7km grid, to redistribute the 7km emissions into the urban polygon and the rest of the grid cell. This sub-7km split is done by population density for SNAP 2 and 7 except heavy duty trucks.

Ambient PM_2.5_ calculations outside Europe follow a very similar approach, however using slightly different resolution and CTM model versions. Also, they are more explicit in terms of differentiating urban and rural low-level emission sources, as described below.

Base case and reduction simulations (15% reduction runs for pollutants PPM total, PPM low-level (SNAP 2+7), SO_2_, NO_X_, NH­_3_, VOC, with met year 2015) have been run with the EMEP CTM at 0.5⁰ resolution, with either an Asia-wide domain as used in the UNEP-CCAC Assessment of Air Pollution in Asia and the Pacific (Hong et al. 2019), or a global domain for all other regions

Transfer coefficients for source regions $j$, source pollutants $p$, to 0.5⁰ grid cells $i$ are defined as

$$\pi_{i}^{pj}=\frac{\Delta\left[ \mathrm{PM}_{2.5} \right]_{i}}{\Delta E_{\mathrm{pj}}}$$

with $p\in\{P,PL,S,N,V\}$ and

$$\Delta\left[ \mathrm{PM}_{2.5} \right]_{i}=\left[ \mathrm{PM}_{2.5} \right]_{base}-\left[ \mathrm{PM}_{2.5} \right]_{red}$$

the difference between base case ($base$) and reduction case ($red$) in modelled PM_2.5_ concentrations in grid cell $i$ when emissions $E$ of pollutant $p$ from source region $j$ are changed. For easier reading, we use upper indices on $\pi$ for sources and lower indices for receptor grid cells.

The 0.5⁰ resolution is not sufficient to capture local gradients of ambient PM_2.5_, which are mostly related to near-ground emissions from sources such as traffic, residential combustion, and waste burning. Therefore, on top of these ordinary transfer coefficient calculations, two global simulations with 0.1⁰ resolution were conducted for met year 2015: a base case simulation, and a simulation in which all residential emissions from located urban areas (all pollutants) were reduced by 30% simultaneously. This additional reduction run was used to split the PPM low-level transfer coefficient into urban and non-urban, and to split the SO_2_ and NO_x_ transfer coefficients ($\pi_{i}^{Sj}$ and $\pi_{i}^{Nj}$) into low-level urban and the rest.

The difference in modelled ambient PM_2.5_ concentrations between base case and urban reduction simulation is shown in Figure A1.


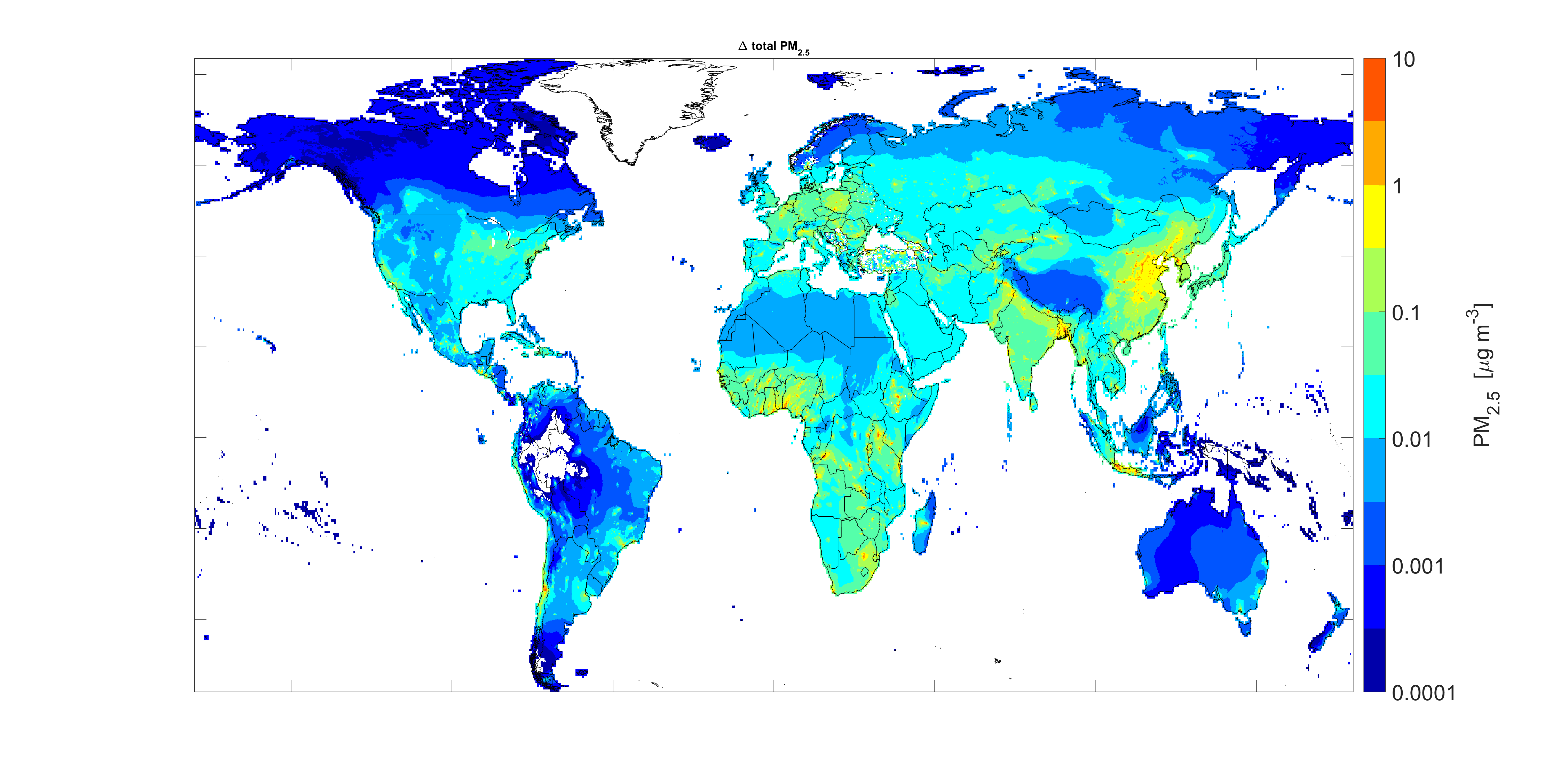


Figure 1. Difference in modelled ambient PM_2.5_ concentrations from a reduction of emissions of all pollutants from the residential sector by 30% in cities globally. Note that the color axis is logarithmic.

While the overall urban reduction simulation allows to identify contributions from cities versus rural areas, the contributions of different source regions are not identified from these two simulations. Here we use the region-specific contributions from low-level sources to the respective 0.5⁰ grid cell to split the effects from urban emission reductions into source regions:

$${\Delta[\mathrm{PPM}_{2.5}]}_{m}^{Lju}=\Delta\left[ \mathrm{PPM}_{2.5} \right]_{m}^{u}\cdot\pi_{i}^{PLj}$$

where $j$ is the source region, $u$ the urban origin, $m$ the 0.1⁰ receptor grid cell, and $i$ the 0.5⁰ grid cell which contains $m$. $\pi_{i}^{j}$ are the transfer coefficients for PPM from low-level sources in region $j$ to grid cell $i$. The corresponding transfer coefficient from low-level urban emissions in region $j$ to grid cell $m$ is then defined by dividing the changes in grid concentrations by the urban PPM emissions from region $j$,

$$\pi_{m}^{PLju}=\frac{\Delta\left[ \mathrm{PPM}_{2.5} \right]_{m}^{ju}}{\Delta E^{Pju}}$$

Since doing a separate reduction simulation for rural low-level emissions at 0.1⁰ resolution was not feasible due to resource constraints, the transfer coefficient for rural low-level PPM emissions is estimated from $\pi_{i}^{PLj}$ and $\pi_{m}^{PLju}$. For this purpose, the residual between urban and total low-level PPM transfer coefficient at the 0.5⁰ resolution is linked to rural low-level PPM emissions to define the equivalent transfer coefficient from rural low-level sources to ambient PM concentrations,

$$\pi_{i}^{PLjr}=\frac{E^{Pj,SNAP2}\pi_{i}^{PLj}-E^{Pj,SNAP2}\pi_{i}^{PLju}}{E^{Pju,SNAP2}}$$

To represent the variability of this transfer coefficient at 0.1⁰ level, we apply the pattern of sub-grid variability of PPM in the 0.1⁰ base case simulation, after subtracting the total contribution from urban low-level emissions estimated as $\left[ \mathrm{PPM}_{2.5} \right]_{base,m}-\left( \left[ \mathrm{PPM}_{2.5} \right]_{base,m}-\left[ \mathrm{PPM}_{2.5} \right]_{red,m} \right)/0.3$.

Equivalently, modelled changes in SO_4_^2-^ from the urban reduction simulation are linked to changes in urban residential SO_2_ emissions to construct an SO_2_ low-level urban transfer coefficient,

$$\pi_{m}^{SLju}=\frac{\Delta\left[ \mathrm{PPM}_{2.5} \right]_{m}^{ju}}{\Delta E^{SLju}}$$

and NO_3_^-^ changes are related to NOx emissions to construct a transfer coefficient from urban low-level NOx emissions,

$$\pi_{m}^{NLju}=\frac{\Delta\left[ \mathrm{NO}_{3}^{-} \right]_{m}^{ju}}{\Delta E^{NLju}}$$

The counterparts $\pi_{i}^{SLjr}$ and $\pi_{i}^{NLjr}$ at 0.5⁰ resolution are estimated equivalently to PPM, however with the total SO_2_ and NO_x_ transfer coefficients used instead of the low-level PPM region-to-grid coefficient. We again apply the spatial pattern of SO_4_^2-^ and NO_3_^-^ variability at 0.1⁰ resolution, after subtracting the estimated contribution from urban low-level sources, to downscale $\pi_{i}^{SLjr}$ and $\pi_{i}^{NLjr}$ to the 0.1⁰ resolution.

A validation of modelled ambient PM_2.5_ for 2015 against global monitoring data from the WHO Ambient Air Pollution Database (WHO 2018) (outside Europe) and the Airbase database (for Europe) (EEA 2018) is shown in Figure S2.


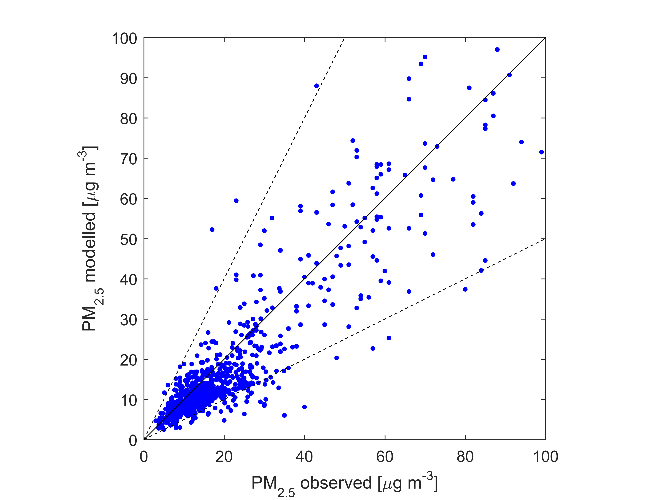


Figure 2. Comparison of modelled PM_2.5_ concentrations for 2015 against monitoring data from the Airbase database (for Europe) and WHO Ambient Air Pollution Database (outside Europe)

# S.3 Population data

Gridded population is taken from publicly available data sets: 100m resolution gridded population from the WorldPop project (worldpop.org.uk, Gaughan et al. 2013) for the Asian countries, and 250m resolution gridded population from the European Commission’s Global Human Settlement project (<https://ghsl.jrc.ec.europa.eu/ghs_pop2019.php> (Schiavina et al. 2019)) for the rest of the world. Classification into cities and rural areas was done by overlaying with city polygons from the Global Rural-Urban Mapping Project (GRUMP, <https://sedac.ciesin.columbia.edu/data/collection/grump-v1> (CIESIN, 2011)). Urban polygons with more than 100,000 inhabitants are taken to identify the localized urban population. Obviously, the sum of the localized city population identified this way is typically smaller than the population classified as urban by census, or the UN World Population Prospects used here. The residual urban population which is not spatially identified is distributed with the rural population outside city polygons.

For future projections, urban and rural population are scaled with the projected trends in urban and rural population in the Medium scenario of the UN World Urbanization Prospects 2018 (UNDESA 2018), starting from the respective base years of the data sets.

Gridded population is used for both the distribution of residential emissions (considering the different prevalence of solid and clean fuels used in urban and rural areas, based on the WHO household energy database, (WHO 2010)), as well as for calculating the population exposure distribution.

# S.4 Emission scenarios

Table 1: Emissions of SO_2_, NO_x_ and PM_2.5_ by world regions. NOC … Without controls; CLE … 2018 legislation; Clean Air: Clean Air scenario (kilotons)

|  | SO_2_ | | | NO_x_ | | | PM_2.5_ | | |
| --- | --- | --- | --- | --- | --- | --- | --- | --- | --- |
|  | 1990 | 2015 | 2040 | 1990 | 2015 | 2040 | 1990 | 2015 | 2040 |
|  | Europe, N. America, Russia | | | | | | | | |
| NOC | 76511 | 52071 | 33515 | 61283 | 55976 | 48878 | 11105 | 9450 | 9204 |
| CLE | 67295 | 15430 | 8981 | 61090 | 29776 | 16979 | 11111 | 5358 | 4154 |
| Clean Air | 67295 | 15430 | 1807 | 61090 | 29776 | 5085 | 11111 | 5358 | 939 |
|  | East Asia | | | | | | | | |
| NOC | 20368 | 71317 | 64564 | 13912 | 42207 | 47461 | 11454 | 22157 | 24464 |
| CLE | 18965 | 16964 | 9288 | 12940 | 29022 | 16272 | 11454 | 9030 | 5826 |
| Clean Air | 18965 | 16964 | 2458 | 12940 | 29022 | 4153 | 11454 | 9030 | 905 |
|  | South and Southeast Asia | | | | | | | | |
| NOC | 6692 | 15958 | 33466 | 7911 | 19960 | 41890 | 9913 | 15670 | 22296 |
| CLE | 6692 | 12952 | 13332 | 7910 | 18381 | 23698 | 9913 | 12566 | 14234 |
| Clean Air | 6692 | 12952 | 2047 | 7910 | 18381 | 3357 | 9913 | 12566 | 979 |
|  | Latin America | | | | | | | | |
| NOC | 6607 | 8285 | 8038 | 7321 | 14172 | 17611 | 2584 | 3848 | 4525 |
| CLE | 6607 | 3728 | 3061 | 7321 | 10503 | 9741 | 2584 | 2975 | 2697 |
| Clean Air | 6607 | 3728 | 491 | 7321 | 10503 | 1427 | 2584 | 2975 | 286 |
|  | Africa | | | | | | | | |
| NOC | 4964 | 8527 | 9282 | 4912 | 10462 | 15774 | 5546 | 9663 | 12649 |
| CLE | 4964 | 6006 | 3862 | 4912 | 9748 | 13551 | 5546 | 9288 | 11652 |
| Clean Air | 4964 | 6006 | 736 | 4912 | 9748 | 1586 | 5546 | 9288 | 607 |
|  | Other countries | | | | | | | | |
| NOC | 8799 | 12978 | 13408 | 7180 | 13379 | 18353 | 1119 | 2043 | 2886 |
| CLE | 8799 | 8694 | 7336 | 7180 | 9816 | 11559 | 1119 | 1406 | 1684 |
| Clean Air | 8799 | 8694 | 499 | 7180 | 9816 | 1307 | 1119 | 1406 | 197 |
|  | World | | | | | | | | |
| NOC | 123942 | 169136 | 162273 | 102518 | 156155 | 189968 | 41720 | 62831 | 76025 |
| CLE | 113321 | 63774 | 45859 | 101353 | 107246 | 91801 | 41726 | 40622 | 40248 |
| Clean Air | 113321 | 63774 | 8038 | 101353 | 107246 | 16914 | 41726 | 40622 | 3913 |

Table 2: Emissions of NH_3_, VOC and BC by world regions. NOC … Without controls; CLE … 2018 legislation; Clean Air: Clean Air scenario (kilotons)

|  | NH_3_ | | | VOC | | | BC | | |
| --- | --- | --- | --- | --- | --- | --- | --- | --- | --- |
|  | 1990 | 2015 | 2040 | 1990 | 2015 | 2040 | 1990 | 2015 | 2040 |
|  | Europe, N. America, Russia | | | | | | | | |
| NOC | 11889 | 11161 | 12072 | 52840 | 50144 | 46514 | 1519 | 1619 | 1424 |
| CLE | 11616 | 10512 | 11292 | 50221 | 23853 | 19785 | 1522 | 888 | 517 |
| Clean Air | 11616 | 10512 | 3026 | 50221 | 23853 | 10437 | 1522 | 888 | 115 |
|  | East Asia | | | | | | | | |
| NOC | 11456 | 16810 | 19807 | 18636 | 34014 | 38852 | 1573 | 1963 | 1479 |
| CLE | 11457 | 16928 | 19978 | 18506 | 23572 | 20407 | 1573 | 1238 | 551 |
| Clean Air | 11457 | 16928 | 4622 | 18506 | 23572 | 7804 | 1573 | 1238 | 56 |
|  | South and Southeast Asia | | | | | | | | |
| NOC | 9245 | 14380 | 19335 | 15001 | 29480 | 40697 | 1699 | 2270 | 2547 |
| CLE | 9245 | 14422 | 19457 | 14981 | 25518 | 26466 | 1699 | 1984 | 1560 |
| Clean Air | 9245 | 14422 | 7749 | 14981 | 25518 | 6417 | 1699 | 1984 | 118 |
|  | Latin America | | | | | | | | |
| NOC | 5767 | 8558 | 10657 | 10152 | 15622 | 19548 | 395 | 652 | 712 |
| CLE | 5767 | 8614 | 10714 | 10145 | 10166 | 10137 | 395 | 517 | 419 |
| Clean Air | 5767 | 8614 | 2900 | 10145 | 10166 | 4198 | 395 | 517 | 41 |
|  | Africa | | | | | | | | |
| NOC | 4286 | 7777 | 9654 | 12208 | 21329 | 25938 | 940 | 1462 | 2016 |
| CLE | 4286 | 7798 | 9714 | 12205 | 20128 | 22440 | 940 | 1414 | 1811 |
| Clean Air | 4286 | 7798 | 4738 | 12205 | 20128 | 3428 | 940 | 1414 | 84 |
|  | Other countries | | | | | | | | |
| NOC | 1680 | 2652 | 3362 | 9153 | 13370 | 18547 | 239 | 414 | 552 |
| CLE | 1680 | 2701 | 3423 | 9111 | 8227 | 9112 | 239 | 314 | 360 |
| Clean Air | 1680 | 2701 | 1134 | 9111 | 8227 | 5630 | 239 | 314 | 20 |
|  | World | | | | | | | | |
| NOC | 44322 | 61338 | 74886 | 117989 | 163959 | 190097 | 6364 | 8381 | 8729 |
| CLE | 44050 | 60974 | 74578 | 115170 | 111464 | 108348 | 6367 | 6355 | 5218 |
| Clean Air | 44050 | 60974 | 24169 | 115170 | 111464 | 37914 | 6367 | 6355 | 435 |

# References

Amann M, Bertok I, Borken-Kleefeld J, et al (2011) Cost-effective control of air quality and greenhouse gases in Europe: Modeling and policy applications. Environ Model Softw 26:1489–1501. https://doi.org/10.1016/j.envsoft.2011.07.012

Amann M, Purohit P, Bhanarkar AD, et al (2017) Managing future air quality in megacities: A case study for Delhi. Atmos Environ 161:99–111. https://doi.org/10.1016/j.atmosenv.2017.04.041

Bhanarkar AD, Purohit P, Rafaj P, et al (2018) Managing future air quality in megacities: Co-benefit assessment for Delhi. Atmos Environ 186:158–177. https://doi.org/10.1016/j.atmosenv.2018.05.026

CIESIN (2011) Global Rural-Urban Mapping Project, Version 1 (GRUMPv1): Urban Extents Grid

Cofala J, Bertok I, Borken-Kleefeld J, et al (2015) Implications of energy trajectories from the World Energy Outlook 2015 for India’s air pollution. Final IIASA Report. International Energy Agency (IEA), Paris

EEA (2018) AirBase - The European air quality database, version 8. European Environment Agency, Copenhagen, Denmark

Gaughan AE, Stevens FR, Linard C, et al (2013) High Resolution Population Distribution Maps for Southeast Asia in 2010 and 2015. PLoS ONE 8:e55882. https://doi.org/10.1371/journal.pone.0055882

GEA (2012) Global Energy Assessment - Toward a Sustainable Future. Cambridge University Press, Cambridge, UK and New York, NY, USA and the International Institute for Applied Systems Analysis, Laxenburg, Austria

Hong Y-C, Hicks K, Malley C, et al (2019) Air Pollution in Asia and the Pacific: Science-based solutions. http://ccacoalition.org/en/resources/air-pollution-asia-and-pacific-science-based-solutions. Accessed 6 Nov 2018

IEA (2019) World Energy Model Documentation - 2019 version. International Energy Agency, Paris, France

IEA (2020) World Energy Statistics and Balances. International Energy Agency, Paris, France

Kiesewetter G, Borken-Kleefeld J, Schöpp W, et al (2015a) Modelling street level PM10 concentrations across Europe: source apportionment and possible futures. Atmos Chem Phys 15:1539–1553. https://doi.org/10.5194/acp-15-1539-2015

Kiesewetter G, Schoepp W, Heyes C, Amann M (2015b) Modelling PM2.5 impact indicators in Europe: Health effects and legal compliance. Environ Model Softw 74:201–211. https://doi.org/10.1016/j.envsoft.2015.02.022

Klimont Z, Kupiainen K, Heyes C, et al (2017) Global anthropogenic emissions of particulate matter including black carbon. Atmospheric Chem Phys 17:8681–8723. https://doi.org/10.5194/acp-17-8681-2017

Menut L, Bessagnet B, Khvorostiyanov D, et al (2013) CHIMERE 2013: a model for regional atmospheric composition modelling. Geosci Model Dev 6:981–1028

Purohit P, Amann M, Mathur R, et al (2010) GAINS-Asia. Scenarios for cost-effective control of air pollution and greenhouse gases in India. International Institute for Applied Systems Analysis (IIASA), Laxenburg, Austria

Rafaj P, Kiesewetter G, Gül T, et al (2018) Outlook for clean air in the context of sustainable development goals. Glob Environ Change 53:1–11. https://doi.org/10.1016/j.gloenvcha.2018.08.008

Rafaj P, Schöpp W, Russ P, et al (2013) Co-benefits of post-2012 global climate mitigation policies. Mitig Adapt Strateg Glob Change 18:801–824. https://doi.org/10.1007/s11027-012-9390-6

Schiavina M, Freire S, MacManus K (2019) GHS population grid multitemporal (1975, 1990, 2000, 2015) R2019A. European Commission, Joint Research Centre (JRC). DOI: 10.2905/42E8BE89-54FF-464E-BE7B-BF9E64DA5218 PID: http://data.europa.eu/89h/0c6b9751-a71f-4062-830b-43c9f432370f

Simpson D, Benedictow A, Berge H, et al (2012a) The EMEP MSC-W chemical transport model – technical description. Atmos Chem Phys 12:7825–7865. https://doi.org/10.5194/acp-12-7825-2012, 2012

Simpson D, Benedictow A, Berge H, et al (2012b) The EMEP MSC-W chemical transport model–technical description. Atmos Chem Phys 12:7825–7865

Tatem AJ, Noor AM, von Hagen C, et al (2007) High Resolution Population Maps for Low Income Nations: Combining Land Cover and Census in East Africa. PLoS ONE 2:e1298. https://doi.org/10.1371/journal.pone.0001298

UNDESA (2018) World Urbanization Prospects: The 2018 Revision. United Nations Department of Economic and Social Affairs., New York

WHO (2018) WHO Global Ambient Air Quality Database (update 2018). World Health Organization (WHO), Geneva, Switzerland

WHO (2010) WHO Household Energy Database. World Health Organisation, Geneva, Switzerland
